# Managing diabetes mellitus using information technology: a systematic review

**DOI:** 10.1186/s40200-015-0174-x

**Published:** 2015-06-03

**Authors:** H. Riazi, B. Larijani, M. Langarizadeh, L. Shahmoradi

**Affiliations:** School of Allied Medical Sciences, Tehran University of Medical Sciences, Tehran, Iran; Endocrinology and Metabolism Research Center, Tehran University of Medical Sciences, Tehran, Iran; School of Health Management and Information Sciences, Iran University of Medical Sciences, Tehran, Iran

**Keywords:** Diabetes mellitus, Systematic review, Medical informatics, Information Technology, Intervention

## Abstract

**Objective:**

To review published evidences about using information technology interventions in diabetes care and determine their effects on managing diabetes.

**Design:**

Systematic review of information technology based interventions.

**Research design and methods:**

MEDLINE®/PubMed were electronically searched for articles published between 2004/07/01 and 2014/07/01. A comprehensive, electronic search strategy was used to identify eligible articles.

Inclusion criteria were defined based on type of study and effect of information technology based intervention in relation to glucose control and other clinical outcomes in diabetic patients. Studies must have used a controlled design to evaluate an information technology based intervention.

A total of 3613 articles were identified based on the searches conducted in MEDLINE from PubMed. After excluding duplicates (*n* = 6), we screened titles and abstracts of 3607 articles based on inclusion criteria. The remaining articles matched with inclusion criteria (*n* = 277) were reviewed in full text, and 210 articles were excluded based on exclusion criteria. Finally, 67 articles complied with our eligibility criteria and were included in this study.

**Results:**

In this study, the effect of various information technology based interventions on clinical outcomes in diabetic patients extracted and measured from selected articles is described and compared to each other.

**Conclusion:**

Information technology based interventions combined with the usual care are associated with improved glycemic control with different efficacy on various clinical outcomes in diabetic patients.

## Introduction

According to International Diabetes Federation (IDF) report, Diabetes Mellitus is a pervasive chronic disease affects 382 million people worldwide and more than 592 million people will be affected within a generation. However most of those cases would be preventable [1].

Diabetic patients with poor blood glucose control have higher mortality and morbidity rate which is related to chronic complications such as neuropathy. Diabetes is a leading cause of death due to increased risk of coronary artery disease and stroke [2].

The estimated total cost of diabetes care in the world was at least USD 548 billion in 2013. This estimation is expected to be more than USD 627 billion for 2035 [1].

A number of information technology based interventions were applied to enhance blood glucose monitoring and diabetes management. Previous evidence demonstrates that information technology can improve diabetes management through better metabolic control and help in the global care of diabetic peoples with chronic illnesses [3–5].

As Marcolino et al. presented in their systematic review and meta-analysis, Telemedicine was associated with a statistically significant and clinically relevant decline in HbA1c level compared to control unlike LDL and blood pressure reduction [6]. Also, according to Siriwardena et al.’s study, Telemedicine appears to be a promising alternative to conventional therapy in diabetic care [7].

Adaji et al. performed a literature review about the use of information technology to enhance diabetes management. They concluded that promoting a productive and informative interaction between the patient and the care team by using information technology based interventions can lead to improve diabetes care [8].

Information technology based interventions have some advantages such as reducing medical errors, generating potential data for research, and increasing the ability for continuous improvement. On the other hand, higher cost of initially and maintenance activities, difficulty of using computer and information systems for healthcare providers and spending more time than interacting with a patient are some disadvantages of using information technology in diabetes care [9, 10].

In this regards, there are some questions: Which intervention is more effective on managing diabetes especially on HbA1C reduction? Is there any relation between type of diabetes and effect of specific intervention? What are the style and variation of interventions in previous studies of using information technology for managing diabetes?

This study was designed in order to perform a comprehensive review of information technology based interventions in diabetes care domain. The purpose of present study was to review published evidences about using information technology in diabetes care and determine the effect of interventions on managing diabetes, includes HbA1C reduction and other clinical outcomes.

## Research design and methods

### Search methods

A literature search was performed in September 2014 using MEDLINE/PubMed database to identify relevant studies published in last ten years from 2004/07/01 to 2014/07/01.

Combination of the following MESH terms and keywords (all fields) were used:

- (("Diabetes Mellitus"[Mesh]) or (Diabetic)) and (("Medical Informatics"[Mesh]) or ("information system") or ("mobile health") or ("electronic health") or ("electronic patient record") or ("electronic medical record") or ("information technology") or ("decision support system") or ("diabetes registry") or ("computerized physician order entry") or ("computerized provider order entry") or ("information network") or ("computer aided diagnosis") or ("computer aided therapy") or ("communication technology") or (telemedicine) or (telehealth) or (sms) or ("short message service") or (telenursing) or (telecare) or (teleradiology))

We did not set another search limits based on study design, study outcome, language or peer-reviewed journals. References of identified articles were also searched for potential articles.

### Inclusion criteria and study selection

Inclusion criteria were defined based on type of study and beneficial or harmful effect of information technology based intervention in relation to glucose control and other clinical outcomes in diabetic patients. The full text of article must be exists and accessible.

Type of information technology based interventions included in this review were as the follows: (1) Telephone coaching, (2) Clinical Decision Support System, (3) Electronic Medical Record/Electronic Health Record, (4) Distance Learning, (5) Computerized Insulin Dose Adjustment, (6) Personal Health Record, (7) Mobile Health/Short Message Service, (8) Telemedicine/Telehealth.

Interventions must aim to improve or promote diabetic care using any information technology based solutions including: (1) interventions designed to improve treatment, monitoring, and management of diabetes. (2) interventions to deliver treatment, education or other diabetes management programs to patients.

We classified the studies according to the hierarchy of study designs developed by the University of California San Francisco Stanford evidence-based practice center and implemented by Kaushal et al. [11] into the following items: Randomized controlled trial (RCT), Non-randomized controlled trial (NRCT), Observational study with control (OS), Observational study without control (OSWC).

Studies must have used a controlled design to evaluate an information technology based intervention. We included all of RCTs, NRCTs and Observational studies with control.

Original articles included in this study. On the other hand, letters (*n* = 2), opinion papers (*n* = 10), reviews and studies that reported preliminary data of another included study (*n* = 46) were not included.

Titles and abstracts of identified articles were screened based on inclusion criteria described above. Full texts of potentially eligible articles were then reviewed. Two of reviewers independently did review, coding and abstracting information from each article. Any discrepancies between the reviewers were resolved through discussion and reference to the original articles.

Exclusion criteria were as follows: (1) case reports or case series with fewer than 10 patients, (2) studies with less than 3 months of follow-up and (3) information technology is not the primary intervention component or information technology based intervention had not implemented (e.g., study protocols).

We did not exclude children with diabetes or pregnant women with gestational diabetes.

**Fig. 1:**
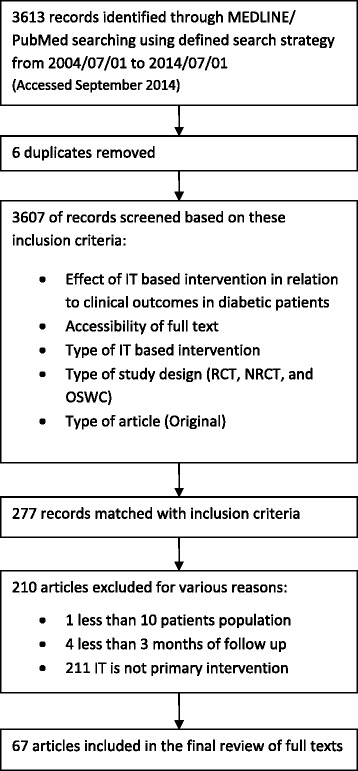
Flow chart of study selection

As shown in Fig. 1, a total of 3613 articles were identified based on the searches conducted in MEDLINE from PubMed. After excluding duplicates, we screened titles and abstracts of 3607 articles based on inclusion criteria. (Kappa agreement index = 0.73).

The remaining articles matched with inclusion criteria (*n* = 277) were reviewed in full text, and 210 articles were excluded based on exclusion criteria. Finally, 67 articles complied with our eligibility criteria and were included in this study. (Kappa agreement index = 0.81).

### Quality assessment

As described above, studies had not control group, population with less than 10 patients or with less than 3 months of follow up, were excluded.

Out of the remained studies, 52 items were randomized controlled trials and three of studies were non randomized controlled trials. Another 11 studies were observational study with control.

### Data extraction

Titles and abstracts of all selected studies were reviewed independently by two reviewers. Papers identified as relevant or of uncertain relevance based on the abstracts were further independently evaluated by both reviewers. Any discrepancies between the two reviewers were resolved by discussion. Reasons for exclusion were documented according to the exclusion criteria.

The data extraction and quality assessment of the studies were performed by the first author and individually checked by second one for accuracy and to identify missing information.

A data extraction form was developed, piloted, and used to extract data which was a modified version of the template form suggested by the Center for Review and Dissemination guidance for systematic review [12]. This form contains following items:

1. article properties e.g. title and UID;
2. study attributes e.g. population, duration of follow, mean age and gender;
3. research type include RCT, NRCT, Other Observational study with control and Observational study without control;
4. intervention type according to the description of inclusion criteria;
5. diabetes type (I, II, GDM, unknown or mixed); and
6. outcomes according to the American diabetes association recommendations for diabetes management [13].

All of extracted data were organized into a single spreadsheet for easier analysis.

### Data-synthesis and analyses

Meta-analysis was not carried out because of the variability of the outcome measures and the heterogeneous nature of the interventions. Therefore, a narrative synthesis carried out based on the interventions, and textual description clustered on the basis of outcome.

The outcomes were effect on the following items:

1. Glycaemic control (changes in HbA1c as the main indicator of treatment effectiveness in diabetic patients [13], and effect on patient self-monitoring of blood glucose (SMBG) or frequency of blood glucose testing)
2. pharmacological and overall approaches to treatment
3. diet and healthy eating
4. DSMS (Diabetes self-management education and support)
5. physical activity
6. blood pressure control (changes in systolic and diastolic blood pressure)
7. lipid management (effect on blood levels of LDL)
8. foot care

## Results

### Description of included studies

Articles were published between 2004/07/01 and 2014/07/01. 12 studies involved a population with type 1 diabetes, and 34 with type 2. A total of 8 studies included a mixed population while type of diabetes was not reported in 13 studies. Table 1 shows the frequency of interventions were applied in studies.

**Table 1:** Frequency of interventions

Abbreviation	Main intervention	No. of studies
TC	Telephone coaching	8
CDSS	Clinical Decision Support System	9
EHR	Electronic Medical Record/Electronic Health Record	7
DL	Distance Learning	7
CIDA	Computerized Insulin Dose Adjustment	1
PHR	Personal Health Record	2
mHealth	Mobile Health/Short Message Service	12
TM	Telemedicine/Telehealth	21
	Summation	67

The summation of total diabetic patients were participated in 67 studies that included in final review was 51,155 persons (mean = 786, median = 137) and the mean age of them was 59.5 years. The proportion of male gender in this population was 46 % and the mean duration of follow was 14 months.

Results are described mainly based on outcomes in regard to the recommendations for managing diabetes noted by the American diabetes association [13]. Below, we describe the effects per outcome group for all included studies. Table 2 shows a summary of all reported measured effects of these 67 studies where results are reported separately by outcome group and study design.

**Table 2:** Summary of measured effects

Outcome category	Study design	Total	Positive effect	Percent	No effect	Percent	Effective interventions	Ineffective interventions
(1) Glycaemic control	RCT	43	29	70.2 %	14	29.8 %	• CIDA	• Telephone coaching
	NRCT	3	1		2		• Distance Learning	• EHR
	OC	11	10		1		• Telemedicine	
(2) Pharmacological and overall approaches to treatment	RCT	3	3	83.3 %	-	16.7 %	• Telemedicine	-
	NRCT	1	1		-		• mHealth	
	OC	2	1		1		• PHR	
							• CDSS	
(3) Diet and healthy eating	RCT	2	1	66.7 %	1	33.3 %	• Telephone coaching	• CDSS
	NRCT	-	-		-		• Telemedicine	
	OC	1	1		-			
(4) Diabetes self-management education and support	RCT	8	6	80 %	2	20 %	• CDSS	• Telephone coaching
	NRCT	-	-		-		• EHR	
	OC	2	2		-		• mHealth	
							• Telemedicine	
(5) Physical activity	RCT	7	5	66.7 %	2	33.3 %	• Telephone coaching	
	NRCT	-	-		-		• Telemedicine	
	OC	2	1		1		• CDSS	
							• mHealth	
(6) Blood pressure control	RCT	16	9	58.8 %	7	41.2 %	• Telephone coaching	• EHR
	NRCT	-	-		-		• CDSS	• mHealth
	OC	1	1		-		• PHR	
							• Telemedicine	
(7) Lipid management (LDL)	RCT	17	9	50 %	8	50 %	• Telephone coaching	• EHR
	NRCT	-	-		-		• CDSS	• PHR
	OC	3	1		2		• Distance learning	• mHealth
							• Telemedicine	
(8) Foot care	RCT	2	1	50 %	1	50 %	• EHR	• CDSS
	NRCT	-	-		-		• mHealth	
	OC	2	1		1			

#### Glycaemic control

Fifty seven studies assessed the effect of information technology strategies on HbA1c. Forty studies (70.2 %) Included 29 RCTs, 1 NRCTs and 8 observational studies with control demonstrated a significant reduction in HbA1c. Other seventeen studies (29.8 %) included 14 RCTs, 2 NRCTs and 1 observational study with control did not find a statistically significant difference between the control and the intervention group with regard to HbA1c reduction. But HbA1c decrease was seen in intervention groups in most studies. It seems most information technology based interventions had a great positive effect on glycaemic control [14–68].

In type I diabetic population, computerized insulin dose adjustment (*n* = 1, 100 % positive effect), and also distance Learning (*n* = 5, 83.3 % positive effect) and Telemedicine/Telehealth (*n* = 14, 82.4 % positive effect) in both type I and type II diabetic population were effective interventions in reducing HbA1c compared with other information technology strategies. On the other hand, telephone coaching and EHR had less effect on HbA1c reduction.

#### Pharmacological and overall approaches to treatment

Six articles were classified in this group. Five studies contained 3 RCTs, 1 NRCT and 1 observational study with control indicated a significant positive changes on medication status for all interventions included CDSS (*n* = 1, 100 % positive effect), PHR (*n* = 1, 100 % positive effect), mHealth (*n* = 1, 100 % positive effect) and Telemedicine/Telehealth (*n* = 3, 66.7 % positive effect). Five of those studies were done in diabetic type II population and 1 was done in both of type I and type II population [14, 17–19, 69].

#### Diet and healthy eating

Three articles include 2 RCTs and 1 observational study with control was classified in this group. Two studies indicated significant positive changes on diet status and healthy eating. Both of them applied a Tele-care intervention. According to those studies, Telephone coaching (*n* = 1) and Telemedicine/Telehealth (*n* = 1) had a positive effect on diabetic patient’s diet [15, 16, 69].

Another study had used a CDSS for patients and providers. This study demonstrated no changes in diet status and healthy eating in type I and type II diabetic population. As noted by authors, delivering the decision support outside of the point of care and being the intervention untimely may be led to obtain negative findings [16].

#### Diabetes self-management education and support (DSMS)

Ten studies assessed the effect of information technology strategies on patient empowerment, knowledge and promoting DSMS. Eight studies (80 %) Included 6 RCTs and 2 observational studies with control demonstrated a significant positive effect on DSMS. Other 2 RCT studies (20 %) did not find a statistically significant difference between the control and the intervention group with regard to effect on DSMS. But increasing patient knowledge was seen in intervention groups in both studies. It seems most information technology based interventions had a great positive effect on DSMS [15, 18, 20, 25, 35, 50, 52, 60, 68].

In both type I and type II diabetic population, Telemedicin/Telehealth (*n* = 4, 100 % positive effect), and mHealth (*n* = 2, 100 % positive effect) and also CDSS (*n* = 1, 100 % positive effect) in type II and EHR (*n* = 1, 100 % positive effect) in type I diabetic population were effective in promoting DSMS and patient knowledge. But telephone coaching has no positive effect on patient empowerment [25, 60].

#### Physical activity

Nine articles were classified in this group. Six studies contained 5 RCTs and 1 observational study with control indicated significant positive changes on patient’s physical activity status and other 3 RCT studies demonstrated no changes [15, 16, 18, 25, 32, 45, 52, 69].

As noted in these studies, telephone coaching (*n* = 2, 50 % positive effect) had a positive effect on physical activity changes only in type II diabetic population. In both type I and type II diabetic population, Telemedicin/Telehealth (*n* = 3, 66.6 % positive effect), CDSS (*n* = 2, 100 % positive effect) and mHealth (*n* = 1, 100 % positive effect) were effective in patient’s physical activity status [25, 69].

#### Blood pressure control (changes in systolic and diastolic blood pressure)

Seventeen studies assessed the effect of information technology strategies on blood pressure control. Ten studies (58.8 %) Included 9 RCTs and 1 observational study with control demonstrated a significant reduction in systolic and diastolic blood pressure. Other 7 RCT studies (41.2 %) did not find a statistically significant difference between the control and the intervention group with regard to blood pressure reduction. Although more marked effects were seen among patients with worse baseline levels [30].

Telephone coaching (*n* = 1, 100 % positive effect), and CDSS (*n* = 3, 66.7 % positive effect) and PHR (*n* = 2, 50 % positive effect) and Telemedicine/Telehealth (*n* = 9, 66.7 % positive effect) were effective interventions in reducing blood pressure compared with other information technology strategies. On the other hand, EHR and mHealth had no effect on blood pressure reduction. It is notable most studies were applied on type II diabetic population [16, 17, 19, 20, 28–30, 32, 35, 41, 44, 47, 50, 55, 59, 67, 70].

#### Lipid management (effect on blood levels of LDL)

Twenty articles include 17 RCTs and 3 observational study with control were classified in this group. Ten studies (52.6 %) include 9 RCTs and 1 observational study with control indicated a significant reduction in LDL levels. Other 10 studies include 8 RCTs and 2 observational study with control did not find a statistically significant difference between the control and the intervention group with regard to blood LDL reduction.

According to those studies, Telephone coaching (*n* = 1, 100 % positive effect), CDSS (*n* = 4, 75 % positive effect), Distance Learning (*n* = 1, 100 % positive effect) and Telemedicine/Telehealth (*n* = 6, 83.3 % positive effect) were effective in reducing LDL levels. On the other hand, EHR, PHR and mHealth had no effect on LDL reduction [16, 17, 19, 28, 29, 32, 39, 41, 43, 46, 47, 49, 55, 57, 59, 63, 64, 67, 71].

#### Foot care

Four studies assessed the effect of information technology strategies on foot care. Two studies Included 1 RCT and 1 observational study with control demonstrated a significant positive effect on foot care status. Other RCT study did not find a statistically significant difference between the control and the intervention group with regard to foot care status.

In type II diabetic population, EHR (*n* = 1, 100 % positive effect) and mHealth (*n* = 1, 100 % positive effect) were effective interventions in foot care compared with other information technology strategies. On the other hand, CDSS had no effect on foot care status in both type I and type II diabetic patients [16, 18, 72].

## Discussion

This systematic review (67 studies, 51,155 patients) indicates that in diabetes patients, information technology based interventions are associated with HbA1c decrease when compared to the usual care alone. HbA1c is a valuable indicator of treatment effectiveness in patients with diabetes, because it is correlated with diabetes complications and reflects average glycaemia over several months [13].

Heterogeneity between studies was not formally assessed and the results of studies (in terms of whether there was a benefit associated with the intervention) were grouped together despite differences in study design, participant characteristics, intervention characteristics and outcomes assessed.

This study has a clearly defined search strategy and study selection method. Therefore, the existence of publication bias cannot be ruled out. We were also not able to carry out a meta-analysis on the impact information technology based interventions have on patient’s clinical status because of heterogeneity and different metrics of reported outcomes.

Our findings demonstrate that distance Learning and Telemedicine/Telehealth in both type I and type II diabetic population were more effective interventions in reducing HbA1c compared with other information technology strategies. Also using new technologies such as Telemedicin/Telehealth and mHealth can improve patient’s knowledge and promote DSMS.

Although previous researches described that elderly peoples have poor technical skills may cause problems in using information technology based interventions and some patients are too busy to use the diabetes self management systems [15], but there is an increase in using of mobile health technologies by patients [73].

Additionally, our review shows that interventions such as Telephone coaching, CDSS, and Telemedicine/Telehealth had a positive impact on LDL and blood pressure.

As noted by Holbrook et al. many primary care providers believed that the technical difficulties with the clinical decision support systems had negative effect on the perceived usefulness of the intervention [44]. Also McMahon et al. expressed a direct relationship between the number of PHR data uploads and larger declines in HbA1c levels [67].

Kim and Song noted that diabetic type II patients benefited from an individualized approach in which the care plan was formulated according to each person’s characteristics [57].

As demonstrated by Sáenz et al. matching information technology with treatment decisions can help providers to obtain better diabetic patients’ health results. On the other hand, computer applications such as decision support systems are useful as an aid for physicians when setting the type and dose of insulin during care period [14].

Also Lowe et al. noted that a template with predefined elements of good wound care in EHR helps providers for measuring and documenting components of good wound care and tracking wound care outcomes [72].

Our findings suggest that information technology based interventions can improve glycaemic control in patients with diabetes and lead to better management of diabetes with different effect of intervention on various clinical findings. Combining multiple information technology based interventions and proposing a comprehensive solution for obtaining better results in various clinical findings lead to better diabetes management may be the suggested future research.

However it seems there is a need to apply some interventions for studying the effect of information technology based interventions on HbA1c and other clinical outcomes in diabetic population.

### Limitations

The articles for this systematic review were selected exclusively from MEDLINE/PubMed based on the search query described. Inaccessibility to the full text of some articles were another limitation in this study.

## Conclusion

A number of health information technology strategies are currently being used to manage diabetes. This systematic review has shown that information technology based interventions combined with the usual care are associated with improved glycemic control with different efficacy on various clinical outcomes in diabetic patients.

The authors stated that there was a distinct need for more comprehensive interventions, in which several technologies were integrated to be able to manage diabetes. Also other randomized studies need to be conducted to evaluate information technologies and their impact on managing diabetes.
